# Metatranscriptomic Reanalysis of Alzheimer’s Brains Identifies Low-Biomass Microbial Signals Including Enrichment of *Acinetobacter radioresistens*

**DOI:** 10.3390/ijms27083430

**Published:** 2026-04-11

**Authors:** Francesc X. Guix

**Affiliations:** Department of Bioengineering, Institut Químic de Sarrià (IQS), Universitat Ramon Llull (URL), 08017 Barcelona, Spain; francesc.guix@iqs.url.edu; Tel.: +34-935-756-081

**Keywords:** Alzheimer’s disease, metatranscriptomics, low-biomass microbiome, Kraken2, Bracken, edgeR, *Acinetobacter*, biofilms, amyloid cross-seeding, oral–brain axis

## Abstract

Alzheimer’s disease (AD) is characterized by progressive cognitive decline and the accumulation of amyloid-β (Aβ) plaques and tau neurofibrillary tangles. Beyond genetic and proteostatic mechanisms, infection- and dysbiosis-based models of AD have gained renewed attention, including the antimicrobial protection hypothesis, in which Aβ may participate in innate immune defense. Here, we reanalyzed ribosomal depleted (Ribo-Zero) RNA-seq data from dorsolateral prefrontal cortex (DLPFC) samples from the Mount Sinai Brain Bank cohort (GSE53697) to screen for non-human transcripts. Reads underwent quality control and adapter trimming, taxonomic classification with Kraken2, abundance re-estimation with Bracken, and differential abundance testing with edgeR. Across 17 samples (9 advanced AD and 8 controls), we detected low-biomass microbial signals, with *Acinetobacter radioresistens* showing enrichment in the AD group (FDR = 0.018). Several additional taxa showed suggestive group differences but did not remain significant after multiple testing correction, including *Lactobacillus iners* (FDR = 0.051). We also performed an exploratory in silico analysis of an *A. radioresistens* biofilm-associated protein homolog, identifying predicted amyloidogenic motifs and surface-exposed regions that may be relevant to cross-seeding hypotheses, although no mechanistic inference can be drawn without experimental validation. Given the technical challenges of inferring microbial signals from post-mortem brain RNA-seq data, including contamination risk, low microbial biomass, and overwhelming host background, these findings should be interpreted as hypothesis-generating and warrant orthogonal validation in larger, microbiome-aware cohorts.

## 1. Introduction

Alzheimer’s disease (AD) is the most common cause of dementia and is neuropathologically defined by extracellular amyloid-β (Aβ) plaques and intracellular neurofibrillary tangles composed of hyperphosphorylated tau. The amyloid hypothesis has provided a productive framework for biomarkers and therapeutics, and recent anti-amyloid antibodies (e.g., lecanemab and donanemab) can slow decline in early symptomatic disease, but the clinical benefit remains modest and does not fully explain disease initiation or heterogeneity [1,2,3]. Large genetic studies implicate pathways beyond Aβ, including innate immunity, lipid metabolism, endocytosis, and barrier function, consistent with a multifactorial etiology in which upstream triggers interact with host susceptibility [4].

Among proposed upstream triggers, infection- and dysbiosis-based models have gained renewed attention. Neurotropic viruses (notably herpesviruses) have been linked to AD risk and pathology in multiple experimental and epidemiological studies, although findings are heterogeneous across cohorts and analytical pipelines [5,6,7,8]. Independently, periodontal disease and oral pathobionts, including *Porphyromonas gingivalis*, have been associated with neuroinflammation and AD-related pathology, supporting an oral–brain axis model in which chronic mucosal infection and systemic inflammation contribute to neurodegeneration [9,10,11].

Mechanistically, the antimicrobial protection hypothesis proposes that Aβ is induced as part of innate immune defense and can entrap microbes, implying that chronic or repeated infection could drive pathological Aβ deposition [12,13,14]. In parallel, ‘cross-seeding’ models suggest that microbial amyloids and biofilm components can accelerate aggregation of host amyloidogenic proteins and amplify neuroinflammation [15,16]. Recent natural experiments and large-scale observational analyses reporting reduced dementia risk after herpes zoster vaccination further reinforce a potential role for immune–pathogen interactions in dementia trajectories [17,18].

Direct characterization of microbial nucleic acids in human brain tissue remains technically challenging. Post-mortem brain is a low-biomass environment in which signals can be dominated by reagent and laboratory contamination, index hopping, and environmental exposure, requiring stringent experimental controls and transparent reporting [19,20,21]. Nonetheless, reanalysis of deep sequencing datasets offers an opportunity to generate testable hypotheses about microbial signatures and their association with neuropathology—particularly when ribosomal depletion (rather than poly(A) selection) improves retention of bacterial transcripts.

Here, we mined ribosomal-depleted RNA-seq data from dorsolateral prefrontal cortex (DLPFC) samples in the Mount Sinai Brain Bank cohort (GSE53697) to (i) identify taxa whose assigned transcript counts differ between advanced AD and controls, and (ii) explore whether a candidate AD-enriched taxon encodes proteins with predicted amyloidogenic segments that could plausibly participate in cross-seeding. We emphasize that this study is exploratory and intended to motivate targeted validation in independent cohorts and with orthogonal assays.

## 2. Results

The study cohort included 17 publicly available post-mortem dorsolateral prefrontal cortex (DLPFC) RNA-seq samples from GSE53697, comprising 9 advanced AD cases and 8 controls, derived from the Mount Sinai Brain Bank (MSBB) cohort [22] (Table 1).

### 2.1. Sequencing Depth and Library Size Distribution

**Figure 1:**
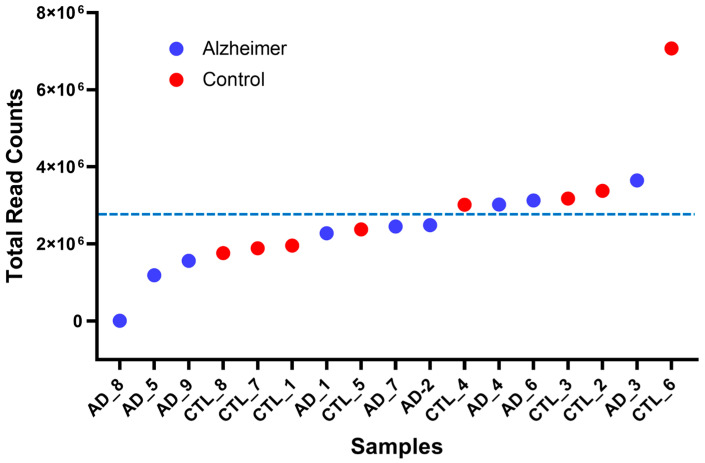
Library size distribution of reads assigned to microbial taxa across the study cohort. Blue points indicate AD samples and red points indicate controls. The dashed line indicates the mean library size.

Following adapter trimming and quality filtering, the remaining library sizes were inspected to confirm that sufficient data were retained for downstream microbial profiling. Figure 1 shows the distribution of reads assigned to microbial taxa across the study cohort after Kraken2 classification and Bracken re-estimation. Although library size varied across samples, as expected for archival post-mortem tissue datasets, all samples retained sufficient depth for downstream analysis. The handling of library size differences for statistical testing is described in Section 4.5.

### 2.2. Global Microbial Composition and Host Contamination

**Figure 2:**
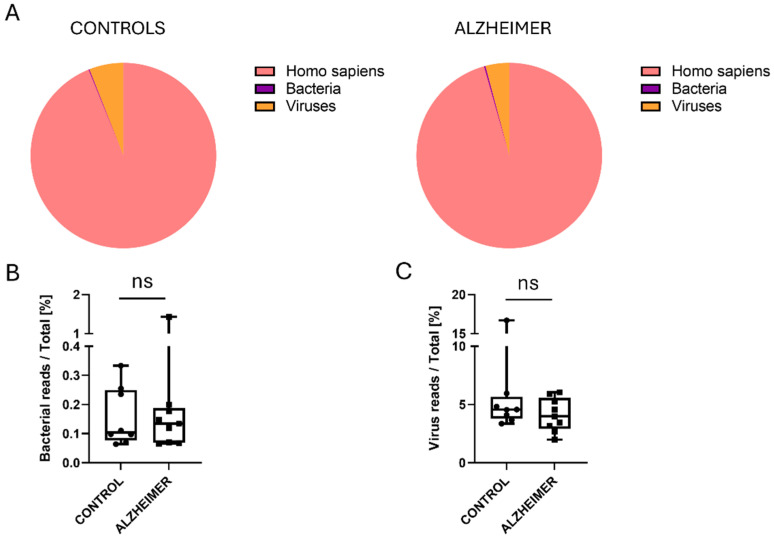
Global composition of assigned reads and relative bacterial/viral signal in AD versus controls. (**A**) Pie charts show the proportion of reads assigned to Homo sapiens, bacterial taxa, and viral taxa in control and Alzheimer’s disease (AD) samples after Kraken2 classification and Bracken re-estimation. (**B**) Bacterial reads as a percentage of total reads in each sample. (**C**) Viral reads as a percentage of total reads in each sample. Box-and-whisker plots show the median and range (min to max), and all individual samples are displayed. All samples, including extreme values, were retained in the statistical analyses. Group comparisons were performed using a two-tailed exact Mann–Whitney test. No significant differences were observed between control and AD samples for either bacterial read fraction (*p* > 0.9999) or viral read fraction (*p* = 0.3704). “ns” indicates no statistically significant difference. Circles and squares represent individual data points for each group.

We next examined the overall taxonomic composition of the aligned reads to understand the signal-to-noise ratio in brain tissue RNA-seq. As expected for human tissue, the vast majority of reads (>99%) mapped to the host genome (*Homo sapiens*). After filtering out host and eukaryotic reads (including fungi and protozoa) to focus specifically on the prokaryotic and viral microbiome, we analyzed the relative proportions of the remaining microbial signal. Figure 2 summarizes the global composition, showing the proportion of bacterial (Figure 2A,B) and viral (Figure 2A,C) reads across the cohort. We observed no statistically significant difference in the total fraction of bacterial or viral reads between the AD and Control groups (Figure 2B,C), suggesting that the disease state is not characterized by a massive, non-specific microbial overgrowth, but rather by subtle shifts in specific taxa.

### 2.3. Differential Abundance Analysis Reveals an Acinetobacter Signature

**Figure 3:**
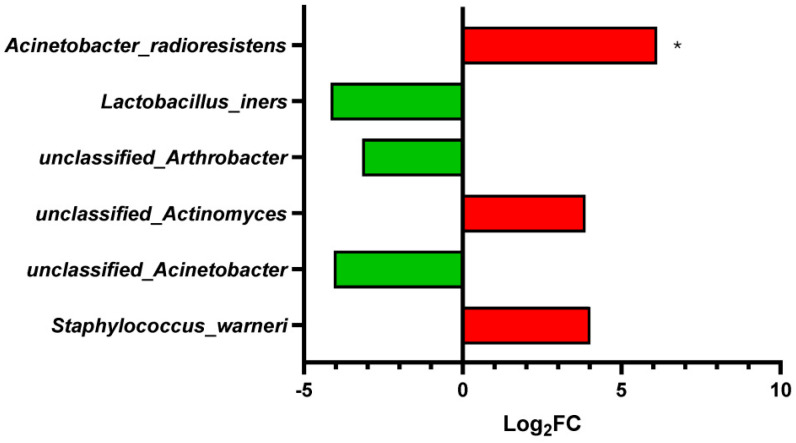
Differentially abundant microbial taxa between Alzheimer’s disease (AD) and control samples identified by edgeR. Species-level differential abundance analysis was performed on the filtered Bracken-derived count matrix using edgeR within MicrobiomeAnalyst. Positive log_2_ fold change (log_2_FC) values (red bars) indicate relative enrichment in the AD group, whereas negative values (green bars) indicate relative enrichment in the control group. Only taxa with FDR < 0.10 are shown in this figure. *Acinetobacter radioresistens* was the only taxon remaining significant after multiple-testing correction at FDR < 0.05 (marked with an *), while the remaining taxa represent suggestive trends. Additional top-ranked taxa not meeting the FDR < 0.10 trend threshold are shown in Supplementary Figure S1 and Supplementary Table S1.

To identify taxa contributing to the differences between Alzheimer’s disease and control samples, we performed differential abundance analysis with edgeR on the filtered species-level count matrix. This analysis identified a limited but detectable microbial shift associated with AD (Figure 3).

The strongest and only FDR-significant association was the enrichment of *Acinetobacter radioresistens* in Alzheimer’s disease samples (log_2_FC = 6.1171; FDR = 0.01791). Several additional taxa showed suggestive differences but did not remain significant after multiple-testing correction, including *Lactobacillus iners*, unclassified *Arthrobacter*, unclassified *Actinomyces*, unclassified *Acinetobacter*, and *Staphylococcus warneri* (Table 2). Additional top-ranked taxa not meeting the FDR < 0.10 trend threshold are shown in Supplementary Table S1, whereas Supplementary Table S2 reports the sample-level raw Bracken counts and group-wise prevalence for the top-ranked taxa to facilitate assessment of sparsity and count distribution across samples.

Overall, these results indicate that the differential signal was driven primarily by *A. radioresistens*, while the remaining taxa should be interpreted as suggestive trends rather than statistically significant findings. This pattern is compatible with differences in the composition of the low-biomass microbial signal between AD and control samples, although this interpretation should remain cautious given the sparse and contamination-prone nature of brain-derived sequencing data.

### 2.4. Exploratory In Silico Analysis of a Bap-like Protein Homolog in A. radioresistens

To explore whether the AD-enriched taxon encodes proteins with potential amyloid-forming properties, we analyzed an Ig-like domain-containing protein from *A. radioresistens* (Accession XDO94130.1), a large surface protein known to form functional amyloids in *Acinetobacter* species. It was identified by BLAST (BLAST+ 2.17.0) as homologous to the biofilm-associated Ig-like repeat protein Bap from *Acinetobacter baumannii*, sharing 54% amino-acid identity.

**Figure 4:**
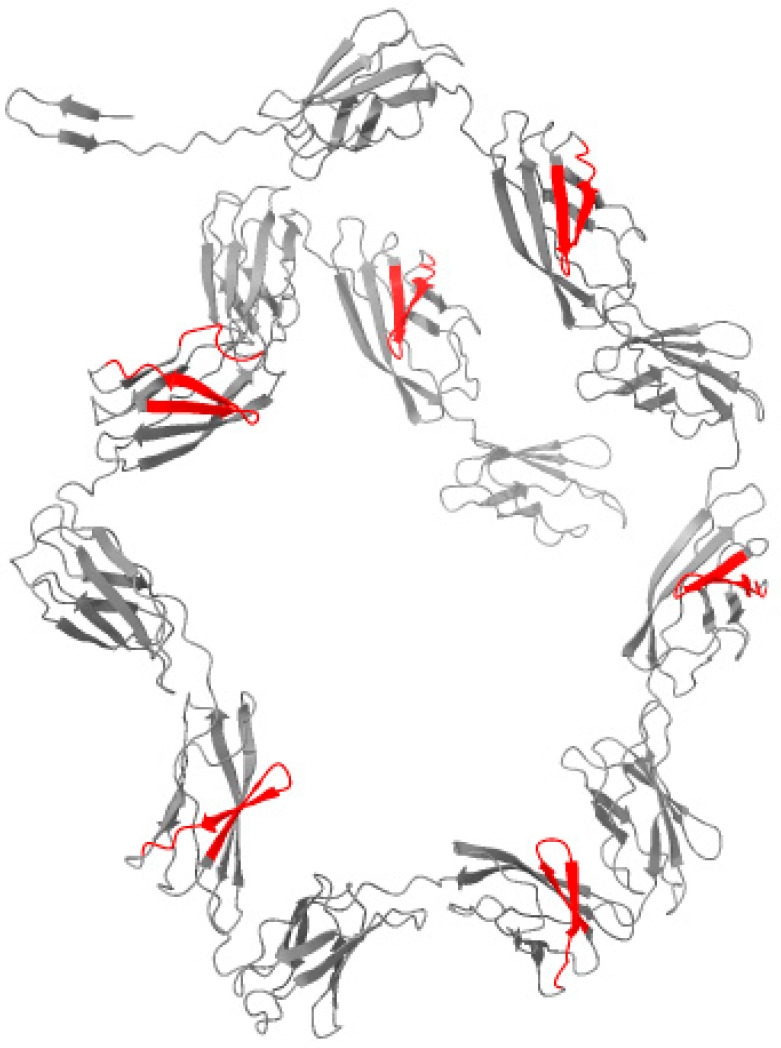
AlphaFold2 structural model of the analyzed bacterial protein region. The protein is shown as a grey ribbon/cartoon representation. The model represents 1120 amino acids and includes a BAP-like domain. Predicted amyloidogenic segments (Waltz) are highlighted in red on a grey surface representation.

Using the Waltz algorithm, we identified 42 distinct hexapeptide regions with high amyloidogenic propensity within the N-terminal domain of the protein (1120 aa region, Table 3). To visualize the spatial arrangement of these regions, we generated a 3D structural model using AlphaFold2. The resulting structure (Figure 4) displays a beta-solenoid fold, a characteristic architecture of bacterial functional amyloids. Crucially, mapping the Waltz-positive segments (highlighted in red) onto the surface representation revealed that several amyloidogenic patches are solvent-exposed. Taken together, these predictions identify candidate amyloidogenic and surface-exposed features that may be relevant to cross-seeding hypotheses, but they do not provide experimental evidence of interaction with Aβ or tau.

## 3. Discussion

Our reanalysis of ribosomal-depleted brain RNA-seq data identified low-biomass microbial signals that differed between advanced AD and control DLPFC samples, with *Acinetobacter radioresistens* showing the strongest association with AD status. These findings align with broader interest in infection- and dysbiosis-based models of AD, including proposals that innate immune activation, barrier dysfunction, and chronic microbial exposures may interact with host genetics to shape neurodegenerative trajectories [4,13]. At the same time, the analytical context—post-mortem tissue and re-purposed RNA-seq—requires a conservative interpretation of taxonomic assignments and effect sizes. The small cohort size and low-biomass nature of the dataset limit statistical power and make it difficult to distinguish weak biological signals from background variability or residual technical effects. In addition, the present analysis is restricted to advanced AD, a single cortical region (DLPFC), and post-mortem tissue, and therefore does not address early or preclinical AD, regional heterogeneity across the brain, or microbial signals in living patients.

The brain is a low-microbial-biomass environment, and multiple studies have demonstrated that reagent and laboratory contaminants can dominate sequencing-based microbiome profiles when true biomass is near the limit of detection [19,20,21]. Several taxa detected here (Table 2 and Table S1)—including environmental actinobacteria (e.g., *Nocardioides*, *Arthrobacter*) and skin-associated organisms—are plausible contaminants introduced during tissue handling, library preparation, or sequencing. Conversely, *A. radioresistens* and *Staphylococcus warneri* are recognized opportunists that can colonize humans and persist on environmental surfaces, complicating source attribution. The restriction to the same tissue region and public cohort, together with consistent processing samples reduce (but do not eliminate) the likelihood that differential signals arise solely from batch artifacts; nevertheless, future work should incorporate extraction blanks, library blanks, and computational decontamination to explicitly model contamination structure. In the absence of microbiome-specific negative controls, retrospective contaminant modeling was not possible, and the biological origin of individual taxa cannot be assigned with confidence.

Notably, some taxa showed relative enrichment in controls, including *Lactobacillus iners* (Figure 3), although this signal did not remain significant after multiple-testing correction and should therefore be interpreted cautiously. *Lactobacillus* species are often discussed as mucosal commensals with potential roles in colonization resistance and immune modulation [23], although *L. iners* is considered atypical within the genus and has been associated with both health- and dysbiosis-related states depending on the ecological context [24,25]. It has also been reported to produce predominantly the L isomer of lactate, with limited D-lactate production [25]. Because L-lactate has recognized signaling and metabolic roles in the CNS [26,27], this observation may be of conceptual interest. However, given the low-biomass nature of the present dataset and the lack of functional or metabolomic measurements, no inference can be made regarding microbial metabolite production in brain tissue. At most, these findings may be viewed as compatible with the possibility that control-enriched taxa reflect a different low-abundance microbial signal rather than a simple global increase in microbial reads in AD.

*Nocardioides* belongs to the *Actinobacteria*, a phylum characterized by substantial metabolic versatility and secondary metabolite production [28], and members of this genus are primarily known as environmental specialists involved in biodegradation [29]. In our dataset, the apparent relative enrichment of *Nocardioides* in controls should be interpreted with caution, particularly given the low-biomass nature of brain RNA-seq microbial profiling and the fact that this signal did not meet the predefined FDR < 0.10 trend threshold (Supplementary Table S1). Rather than supporting a specific biological role, this observation is most appropriately interpreted as an exploratory signal that may reflect subtle differences in a sparse microbial background.

*Arthrobacter* spp. were also relatively enriched in controls (Table 2). These organisms are classically described as soil-associated actinobacteria and, in a low-biomass setting, may plausibly reflect environmental carryover or other exogenous sources. Although metabolites derived from *Arthrobacter* metabolism, such as 6-hydroxy-L-nicotine, have shown neurobehavioral effects in experimental animal studies [30,31,32], such observations cannot be extrapolated to the present dataset. We therefore do not infer that *Arthrobacter*-associated metabolites are produced in brain tissue in vivo. Instead, these examples are noted only to illustrate that taxa detected in low-biomass datasets can have biologically interesting metabolic repertoires, even when their relevance to the present samples remains uncertain. Neurovascular unit dysfunction and blood–brain barrier (BBB) breakdown occur early in cognitive decline and are increasingly recognized as contributors to AD pathophysiology [33,34]. BBB impairment could facilitate entry of circulating microbial products (e.g., LPS, peptidoglycan) or rare microbes into perivascular and parenchymal compartments, potentially amplifying microglial activation and inflammasome signaling. However, a purely ‘leakier BBB → more pathogens’ interpretation would predict a higher overall bacterial/viral signal in AD. Consistent with this counter-argument, we observed no significant differences in the fraction of bacterial or viral reads (normalized to total reads per sample) between controls and AD (Figure 2; two-tailed exact Mann–Whitney test). In this framework, microbial signals detected in RNA-seq data may reflect a combination of true biological exposure and sampling artifacts; discriminating these requires spatially resolved assays (e.g., RNAscope, immunohistochemistry) and careful modeling of post-mortem interval effects.

Aβ is increasingly viewed as an innate immune effector with antimicrobial properties, providing a plausible link between chronic infection and plaque deposition [12,13,14]. Separately, bacterial functional amyloids and biofilm matrix proteins can adopt β-rich architectures and have been proposed to promote aggregation of host amyloidogenic proteins via heterologous seeding [15]. Our in silico analysis of a Bap-like protein homolog identified numerous amyloidogenic segments and surface-exposed patches in a predicted structural model. Biofilm-associated proteins with Ig-like repeats have been described as key determinants of adhesion and biofilm formation in *Acinetobacter* spp., supporting the plausibility of amyloid-like motifs in this protein family [35]. While purely computational, these results motivate biochemical experiments to test whether the protein (or its fragments) forms amyloid fibrils and whether it accelerates Aβ aggregation. Inflammation-driven ‘cross-seeding’ is also supported by microglial ASC specks, which can bind Aβ and promote its aggregation in vivo, linking innate immunity to propagative amyloid assembly [16].

The taxa highlighted here do not exclude alternative or complementary microbial signals. Herpesvirus-associated signatures have been reported in several AD brain cohorts [6], although other analyses have questioned the robustness of these associations and emphasized potential analytical confounders [7,8]. Likewise, a growing body of work has explored an oral–brain axis in AD, in which periodontal inflammation and oral dysbiosis correlate with cognitive decline and may, through hematogenous spread or cranial nerve pathways, increase the likelihood that oral taxa or their products reach the CNS [9,10,11,18]. In our dataset, *Actinomyces* spp. were among the taxa showing relative enrichment in AD (Table 2), although this signal did not remain significant after multiple-testing correction and should therefore be interpreted cautiously. *Actinomyces* are common constituents of dental plaque, and in a longitudinal cohort, higher serum IgG to periodontal taxa—including *Actinomyces naeslundii*—was associated with incident AD [36]. Intracranial dissemination of Actinomyces from oral foci has also been clinically documented, including brain abscesses attributed to *Actinomyces meyeri* of presumed oral origin [37], indicating that mouth-to-brain translocation is biologically plausible under some conditions. Consistent with a broader polymicrobial framework, previous sequencing and immunohistology studies of post-mortem AD tissue have reported increased bacterial burden and diverse taxa, including *Staphylococcus* and oral-associated genera, relative to controls [37,38,39]. We also detected *Streptococcus* salivarius and *Staphylococcus warneri* in this dataset (Table 2 and Table S1). *Streptococci* are abundant oral commensals, so their detection is compatible with a possible oral source, although directionality and abundance patterns may vary across cohorts. Coagulase-negative *staphylococci* such as *S. warneri* are opportunists with biofilm-forming capacity and documented invasive potential [40]. Taken together, these observations do not identify a specific causal organism, but they are compatible with the possibility that low-level, polymicrobial exposure may be relevant to neuroinflammatory processes in a subset of cases.

If microbial exposures contribute to AD onset or progression in a subset of individuals, preventive strategies might include vaccination, periodontal disease management, and targeted modulation of mucosal immunity. Natural experiments and emulated target trials have reported lower dementia incidence after herpes zoster vaccination, findings that are compatible with roles for immune training or reduced viral reactivation [17,18]. However, such observational designs remain susceptible to confounding and do not identify specific pathogens. Translational progress will require (i) prospective cohorts with standardized biospecimen handling and microbiome-aware negative controls, (ii) multimodal validation in tissue, including sequencing, proteomics, and imaging, and (iii) experimental studies testing whether candidate microbial products can influence amyloid aggregation or inflammatory signaling under biologically relevant conditions.

Because this study is based on retrospective reanalysis of public RNA-seq data, it cannot provide microbiome-aware negative controls, wet-lab validation, or robust estimates of absolute microbial abundance; these will require prospective experimental studies specifically designed for low-biomass microbiome investigation.

## 4. Materials and Methods

### 4.1. Bioinformatic Workflow and Data Processing

**Figure 5:**
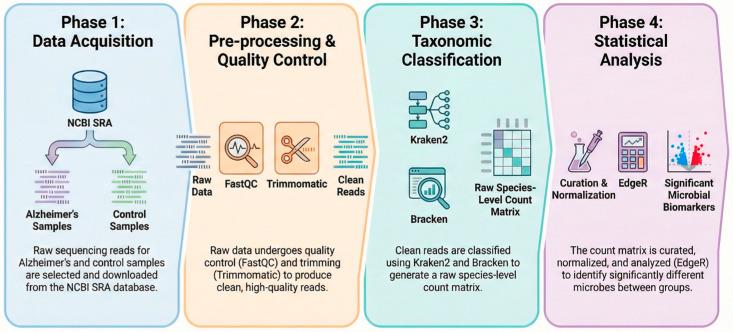
Kraken2 taxonomy and analysis workflow. Raw sequencing reads from Alzheimer’s disease (AD) and control samples were retrieved from NCBI SRA, quality checked (FastQC) and trimmed (Trimmomatic), classified with Kraken2 and abundance re-estimated with Bracken to generate a species-level count matrix, which was curated/normalized and tested for differential abundance (edgeR). Arrows indicate the workflow progression, and colors are used to visually distinguish the different analysis phases.

To systematically identify microbial signatures from non-targeted RNA-sequencing data, we implemented a multi-stage bioinformatic pipeline (Figure 5). Raw sequencing reads from 17 post-mortem DLPFC samples (9 AD, 8 controls) were retrieved from the SRA database. Publicly verifiable metadata for the analyzed subset are summarized in Table 1.

Given the potential for technical artifacts in older datasets, a rigorous quality control phase was prioritized. Initial FastQC (Galaxy wrapper Version 0.74+galaxy1) analysis indicated the presence of Illumina adapters and quality drop-offs at the 3’ ends of reads. Consequently, we applied Trimmomatic (Galaxy wrapper Version 0.39+galaxy2) with a specific sliding window filter (4:20), which discarded reads where the average Phred quality score dropped below 20 in a 4-base window. This preprocessing step was critical to ensure that subsequent taxonomic classification was based on high-confidence sequence data rather than sequencing errors.

### 4.2. Data Acquisition and Cohort Selection

Raw paired-end RNA-seq data were obtained from the NCBI Sequence Read Archive (SRA) corresponding to the Mount Sinai Brain Bank (MSBB) cohort (GSE53697) [22,41]. Using the NCBI SRA Run Selector, we filtered for “Alzheimer’s Disease” and “Control” samples originating from the dorsolateral prefrontal cortex (DLPFC; Brodmann area 46) and prepared using the Ribo-Zero library strategy. This strategy was selected to maximize the retention of non-polyadenylated microbial transcripts. The final dataset comprised 17 samples (9 advanced AD; 8 controls) selected from the same tissue region and public cohort. Publicly verifiable metadata for the analyzed subset are summarized in Table 1. Reads were retrieved from SRA using the Galaxy tool ‘Download and Extract Reads in FASTQ’ (Galaxy wrapper v3.1.1+galaxy1) to ensure efficient retrieval of large datasets.

### 4.3. Preprocessing and Quality Control

All preprocessing steps were executed on the Galaxy platform (Galaxy version 26.0.rc1; usegalaxy.org) [42]. Initial read quality was assessed using FastQC (Galaxy wrapper Version 0.74+galaxy1) [43], focusing on “Per base sequence quality” (Phred scores) and “Adapter Content.” To mitigate sequencing errors and remove technical artifacts, reads were processed with Trimmomatic (Galaxy wrapper Version 0.39+galaxy2) [44] in paired-end mode. We applied the standard Illumina TruSeq3 adapter trimming and a sliding window filtration (Sliding Window: 4:20), which cuts the read once the average quality within a 4-base window falls below a Phred score of 20.

Because this public post-mortem RNA-seq dataset was not generated under microbiome-specific sterile sampling conditions and did not include extraction blanks or library blanks, formal contaminant modeling/decontamination could not be performed. In the absence of microbiome-specific negative controls, retrospective contaminant modeling was not possible, and the biological origin of individual taxa cannot be assigned with confidence. Therefore, all taxonomic signals reported here should be interpreted conservatively as candidate associations rather than definitive evidence of a resident brain microbiome.

### 4.4. Taxonomic Classification

High-quality, cleaned reads were taxonomically classified using Kraken2 (Galaxy wrapper Version 2.17.1+galaxy0) [45]. Abundance re-estimation was performed with Bracken (Galaxy wrapper Version 3.1+galaxy0) [46]. We utilized the PlusPF (17 May 2021) database (Standard plus protozoa & fungi), which is the most comprehensive standard index available in Galaxy. To maximize sensitivity for low-abundance, non-polyadenylated microbial transcripts in a brain tissue context, the Kraken2 confidence threshold was set to 0.0. We acknowledge that this choice prioritizes sensitivity over specificity and may increase the risk of false-positive assignments in low-biomass data. To mitigate over-interpretation, taxonomic abundances were subsequently re-estimated with Bracken, filtered by prevalence and variance, and interpreted conservatively, with all downstream findings treated as candidate associations rather than definitive evidence of a resident brain microbiome. Output options were configured to print scientific names and generate reports with aggregate counts per clade. For preliminary visual exploration of the taxonomic hierarchy, Kraken report files were converted using Krakentools (Galaxy wrapper Version 1.2.1+galaxy0) and visualized as interactive charts using Krona (Galaxy wrapper Version 2.7.1+galaxy0) [47].

### 4.5. Data Matrix Generation and Statistical Analysis

Individual Bracken species reports were merged into a single abundance matrix using the KrakenTools (Galaxy wrapper Version 1.2.1+galaxy2) utility for combining Kraken-style reports. The resulting matrix was exported to Microsoft Excel for manual curation only. Metadata rows and entries lacking species-level information were removed, and all counts assigned to the domain Eukaryota (including *Homo sapiens*) were excluded prior to downstream statistical analysis. For transparency, raw Bracken counts for the top-ranked taxa were compiled at the sample level and are provided together with group-wise prevalence in Supplementary Table S2.

Statistical analysis was performed in the Marker Data Profiling module of Micro-biomeAnalyst 2.0 [48]. The curated species-level count matrix and metadata were up-loaded using the “Taxonomy included” option. Data integrity checks confirmed correct sample matching. Low-abundance and low-variance features were filtered using the platform settings applied in the final analysis (prevalence filter and IQR-based variance filter). For exploratory visualization, library sizes were adjusted by column-sum scaling (equivalent to total sum scaling, TSS). Differential abundance analysis between Alzheimer’s disease and control samples was performed with edgeR (implemented within MicrobiomeAnalyst 2.0, Marker Data Profiling module) [49] on the filtered Bracken-derived count matrix using edgeR’s native normalization framework within MicrobiomeAnalyst. Taxa with a false discovery rate (FDR) < 0.05 were considered statistically significant, whereas taxa with 0.05 ≤ FDR < 0.10 were considered suggestive trends. Graphs were prepared in GraphPad Prism v9.0.0 (GraphPad Software, San Diego, CA, USA). The schematic overview of the bioinformatic workflow (Figure 5) was generated with Gemini 3 (Google) and then curated by the authors.

### 4.6. Structural Modeling and Amyloid Prediction

For the most strongly AD-enriched bacterial species (*Acinetobacter radioresistens*), we identified a candidate protein sequence via NCBI Protein (Accession XDO94130.1), homologous to the biofilm-associated protein (Bap) of *A. baumannii*. Due to the extreme length of the protein (>3000 amino acids), the N-terminal domain (approx. 1000 residues) was selected for modeling. The 3D structure was predicted using AlphaFold2 via the ColabFold notebook version 1.6.1 [50,51]. The model with the highest confidence score (pLDDT, rank_001) was selected for analysis.

Amyloidogenic potential was screened using the Waltz algorithm (https://waltz.switchlab.org/; accessed on 15 January 2026) [52]. The resulting structural model was visualized using UCSF ChimeraX version 1.11 [53]. To map the predicted amyloidogenic regions onto the structure, we utilized the command interface to apply a white background, grey surface rendering, and specific red coloring (color #1:start-end red) for residues identified by Waltz, allowing for the assessment of solvent exposure.

## 5. Conclusions

Reanalysis of ribosomal RNA-depleted brain RNA-seq datasets identified low-biomass microbial signatures that distinguished advanced Alzheimer’s disease (AD) dorsolateral prefrontal cortex (DLPFC) samples from controls, with Acinetobacter radioresistens showing the most consistent association with AD status. These observations complement increasing interest in infection- and dysbiosis-informed frameworks for AD, in which innate immune activation, blood–brain barrier integrity, chronic microbial exposures, and host genetic susceptibility may together shape neurodegenerative trajectories.

As an exploratory analysis based on bulk post-mortem RNA-seq data not originally designed for microbiome profiling, our findings should be interpreted as hypothesis-generating and as a foundation for targeted follow-up studies. Future work using dedicated microbiome sequencing protocols, matched negative controls, spatially resolved approaches, and larger cohorts will be important to refine taxonomic resolution, address potential background signals in low-biomass samples, and clarify questions of microbial viability, localization, and temporal dynamics relative to neuropathology. Such studies will help determine the biological relevance of these signals and further evaluate their potential contribution to AD pathogenesis.

## Figures and Tables

**Table 1:** Publicly retrievable characteristics of the GSE53697 samples included in this reanalysis. The analyzed subset was derived from the Mount Sinai Brain Bank (MSBB) cohort [22]. Only metadata directly supported by the public GEO sample records are shown. Because sample-level age-at-death, post-mortem interval, Braak stage, and related clinical descriptors could not be reliably reconstructed for the selected 17-sample subset from the publicly retrievable metadata files available to us, de novo demographic/statistical comparisons are not presented here. AD: Alzheimer’s disease; DLPFC: dorsolateral prefrontal cortex.

Characteristic	Alzheimer’s Disease (AD)	Control (CTL)
Number of subjects (*n*)	9	8
GEO sample titles	RNAseq_AD_9 to RNAseq_AD_17	RNAseq_Ctrl_1 to RNAseq_Ctrl_8
GEO accession IDs	GSM1885088 to GSM1885096	GSM1885080 to GSM1885087
Disease status (public GEO metadata)	Advanced AD	Control
Tissue	brain	brain
Tissue subtype	DLPFC	DLPFC
Extracted molecule	total RNA	total RNA
Library strategy	RNA-Seq	RNA-Seq
Library preparation	ribominus selection	ribominus selection
Instrument model	Illumina HiSeq 2500	Illumina HiSeq 2500

**Table 2:** Differential abundance results for microbial taxa detected in Alzheimer’s disease (AD) versus control samples. Taxa are ranked by FDR from the edgeR analysis. Positive log_2_FC values indicate relative enrichment in the AD group, whereas negative log_2_FC values indicate relative enrichment in the control group. Taxa with FDR < 0.05 were considered statistically significant, and taxa with 0.05 ≤ FDR < 0.10 were considered suggestive trends.

Bacteria	log_2_FC	*p*-Value	FDR
*Acinetobacter radioresistens*	6.1171	5.76 × 10^−5^	0.01791
*Lactobacillus iners*	−4.1559	3.34 × 10^−4^	0.051994
unclassified *Arthrobacter*	−3.1695	7.78 × 10^−4^	0.080637
unclassified *Actinomyces*	3.8616	1.55 × 10^−3^	0.095283
unclassified *Acinetobacter*	−4.0619	1.62 × 10^−3^	0.095283
*Staphylococcus warneri*	4.0163	1.84 × 10^−3^	0.095283

**Table 3:** Predicted amyloidogenic regions in the *Acinetobacter radioresistens* Bap homolog. Summary of the in silico analysis of the Ig-like domain-containing protein (GenBank Accession: XDO94130.1), identified as a homolog of the biofilm-associated protein (Bap). The Waltz algorithm was used to screen the full-length protein sequence (3436 amino acids) for hexapeptides with high amyloid-forming propensity. A total of 42 distinct amyloidogenic regions were identified; their specific amino acid coordinates are listed in the final column. These segments represent potential sites for structural aggregation and cross-seeding interactions.

Accession Number	Protein Name	Organism	Sequence Length (aa)	No. of Amyloidogenic Regions	Amyloidogenic Region Coordinates
XDO94130.1	Ig-like domain-containing protein	*Acinetobacter radioresistens*	3436	42	135–149; 157–162; 307–321; 329–334; 479–493; 501–506; 651–665; 673–678; 823–837; 845–850; 995–1009; 1017–1022; 1167–1181; 1189–1194; 1339–1353; 1361–1366; 1485–1493; 1571–1578; 1654–1660; 1737–1744; 1839–1847; 1879–1887; 2086–2092; 2169–2175; 2252–2258; 2354–2362; 2394–2402; 2463–2469; 2576–2584; 2603–2609; 2803–2816; 2829–2834; 2837–2845; 2854–2860; 2869–2876; 2950–2958; 2996–3004; 3080–3085; 3113–3118; 3150–3161; 3380–3389; 3431–3436

## Data Availability

All sequencing data analyzed in this study are publicly available from the NCBI Gene Expression Omnibus under accession GSE53697 and associated SRA records. Analysis steps were performed using the Galaxy platform and standard open-source tools as described in the Methods.

## Supplementary Materials

The following supporting information can be downloaded at: https://www.mdpi.com/article/10.3390/ijms27083430/s1.

## Abbreviations

The following abbreviations are used in this manuscript:

SD	Standard Deviation
FC	Fold Change
AD	Alzheimer’s disease
SEM	Standard Error of the Mean

## References

[1] Selkoe D.J., Hardy J. (2016). The amyloid hypothesis of Alzheimer’s disease at 25 years. EMBO Mol. Med..

[2] van Dyck C.H., Swanson C.J., Aisen P., Bateman R.J., Chen C., Gee M., Kanekiyo M., Li D., Reyderman L., Cohen S. (2023). Lecanemab in early Alzheimer’s disease. N. Engl. J. Med..

[3] Sims J.R., Zimmer J.A., Evans C.D., Lu M., Ardayfio P., Sparks J., Wessels A.M., Shcherbinin S., Wang H., Nery E.S.M. (2023). Donanemab in Early Symptomatic Alzheimer Disease: The TRAILBLAZER-ALZ 2 Randomized Clinical Trial. JAMA.

[4] Kunkle B.W., Grenier-Boley B., Sims R., Bis J.C., Damotte V., Naj A.C., Boland A., Vronskaya M., van der Lee S.J., Amlie-Wolf A. (2019). Genetic meta-analysis of diagnosed Alzheimer’s disease identifies new risk loci and implicates Aβ, tau, immunity and lipid processing. Nat. Genet..

[5] Piacentini R., De Chiara G., Li Puma D.D., Ripoli C., Marcocci M.E., Garaci E., Palamara A.T., Grassi C. (2014). HSV-1 and Alzheimer’s disease: More than a hypothesis. Front. Pharmacol..

[6] Readhead B., Haure-Mirande J.-V., Funk C.C., Richards M.A., Shannon P., Haroutunian V., Sano M., Liang W.S., Beckmann N.D., Price N.D. (2018). Multiscale analysis of independent Alzheimer’s cohorts finds disruption of molecular, genetic, and clinical networks by human herpesvirus. Neuron.

[7] Rizzo R. (2020). Controversial role of herpesviruses in Alzheimer’s disease. PLoS Pathog..

[8] Allnutt M.A., Johnson K., Bennett D.A., Connor S.M., Troncoso J.C., Pletnikova O., Albert M.S., De Jager P.L., Jacobson S. (2020). Human herpesvirus 6 (HHV-6) detection in Alzheimer’s disease cases and controls across multiple cohorts. Neuron.

[9] Dominy S.S., Lynch C., Ermini F., Benedyk M., Marczyk A., Konradi A., Nguyen M., Haditsch U., Raha D., Griffin C. (2019). Porphyromonas gingivalis in Alzheimer’s disease brains: Evidence for disease causation and treatment with small-molecule inhibitors. Sci. Adv..

[10] Villar A., Paladini S., Cossatis J. (2024). Periodontal disease and Alzheimer’s: Insights from a systematic literature network analysis. J. Prev. Alzheimer’s Dis..

[11] Sarmiento-Ordóñez J.M., Brito-Samaniego D.R., Vásquez-Palacios A.C., Pacheco-Quito E.M. (2025). Association between Porphyromonas gingivalis and Alzheimer’s disease in older adults: A comprehensive review. Infect. Drug Resist..

[12] Soscia S.J., Kirby J.E., Washicosky K.J., Tucker S.M., Ingelsson M., Hyman B., Burton M.A., Goldstein L.E., Duong S., Tanzi R.E. (2010). The Alzheimer’s disease-associated amyloid β-protein is an antimicrobial peptide. PLoS ONE.

[13] Moir R.D., Lathe R., Tanzi R.E. (2018). The antimicrobial protection hypothesis of Alzheimer’s disease. Alzheimer’s Dement..

[14] Eimer W.A., Vijaya Kumar D.K., Navalpur Shanmugam N.K., Rodriguez A.S., Mitchell T., Washicosky K.J., György B., Breakefield X.O., Tanzi R.E., Moir R.D. (2018). Alzheimer’s disease-associated β-amyloid is rapidly seeded by Herpesviridae to protect against brain infection. Neuron.

[15] Friedland R.P., Chapman M.R. (2017). The role of microbial amyloid in neurodegeneration. PLoS Pathog..

[16] Venegas C., Kumar S., Franklin B.S., Dierkes T., Brinkschulte R., Tejera D., Vieira-Saecker A., Schwartz S., Santarelli F., Kummer M.P. (2017). Microglia-derived ASC specks cross-seed amyloid-β in Alzheimer’s disease. Nature.

[17] Taquet M., Dercon Q., Todd J.A., Harrison P.J. (2024). The recombinant shingles vaccine is associated with lower risk of dementia. Nat. Med..

[18] Eyting M., Xie M., Michalik F., Heß S., Chung S., Geldsetzer P. (2025). A natural experiment on the effect of herpes zoster vaccination on dementia. Nature.

[19] Salter S.J., Cox M.J., Turek E.M., Calus S.T., Cookson W.O., Moffatt M.F., Turner P., Parkhill J., Loman N.J., Walker A.W. (2014). Reagent and laboratory contamination can critically impact sequence-based microbiome analyses. BMC Biol..

[20] Eisenhofer R., Minich J.J., Marotz C., Cooper A., Knight R., Weyrich L.S. (2019). Contamination in low microbial biomass microbiome studies: Issues and recommendations. Trends Microbiol..

[21] Fierer N., Leung P.M., Lappan R., Eisenhofer R., Ricci F., Holland S.I., Dragone N., Blackall L.L., Dong X., Dorador C. (2025). Guidelines for preventing and reporting contamination in low-biomass microbiome studies. Nat. Microbiol..

[22] Wang M., Beckmann N.D., Roussos P., Wang E., Zhou X., Wang Q., Ming C., Neff R., Ma W., Fullard J.F. (2018). The Mount Sinai cohort of large-scale genomic, transcriptomic and proteomic data in Alzheimer’s disease. Sci. Data.

[23] Amabebe E., Anumba D.O.C. (2018). The vaginal microenvironment: The physiologic role of Lactobacilli. Front. Med..

[24] Zheng N., Guo R., Wang J., Zhou W., Ling Z. (2021). Contribution of Lactobacillus iners to vaginal health and diseases: A systematic review. Front. Cell. Infect. Microbiol..

[25] Vaneechoutte M. (2017). Lactobacillus iners, the unusual suspect. Res. Microbiol..

[26] Suzuki A., Stern S.A., Bozdagi O., Huntley G.W., Walker R.H., Magistretti P.J., Alberini C.M. (2011). Astrocyte–neuron lactate transport is required for long-term memory formation. Cell.

[27] Yang J., Ruchti E., Petit J.M., Jourdain P., Grenningloh G., Allaman I., Magistretti P.J. (2014). Lactate promotes plasticity gene expression by potentiating NMDA signaling in neurons. Proc. Natl. Acad. Sci. USA.

[28] Barka E.A., Vatsa P., Sanchez L., Gaveau-Vaillant N., Jacquard C., Klenk H.-P., Clément C., Ouhdouch Y., van Wezel G.P. (2015). Taxonomy, physiology, and natural products of Actinobacteria. Microbiol. Mol. Biol. Rev..

[29] Ma Y., Wang J., Liu Y., Wang X., Zhang B., Zhang W., Chen T., Liu G., Xue L., Cui X. (2023). Nocardioides: “Specialists” for hard-to-degrade pollutants in the environment. Molecules.

[30] Hritcu L., Stefan M., Brandsch R., Mihasan M. (2013). 6-hydroxy-L-nicotine from *Arthrobacter nicotinovorans* sustain spatial memory formation by decreasing brain oxidative stress in rats. J. Physiol. Biochem..

[31] Hritcu L., Stefan M., Brandsch R., Mihasan M. (2015). Enhanced behavioral response by decreasing brain oxidative stress to 6-hydroxy-L-nicotine in Alzheimer’s disease rat model. Neurosci. Lett..

[32] Boiangiu R.S., Mihasan M., Gorgan D.L., Stache B.A., Petre B.A., Hritcu L. (2020). Cotinine and 6-hydroxy-L-nicotine reverse memory deficits and reduce oxidative stress in Aβ25–35-induced rat model of Alzheimer’s disease. Antioxidants.

[33] Montagne A., Barnes S.R., Sweeney M.D., Halliday M.R., Sagare A.P., Zhao Z., Toga A.W., Jacobs R.E., Liu C.Y., Amezcua L. (2015). Blood–brain barrier breakdown in the aging human hippocampus. Neuron.

[34] Nation D.A., Sweeney M.D., Montagne A., Sagare A.P., D’Orazio L.M., Pachicano M., Sepehrband F., Nelson A.R., Buennagel D.P., Harrington M.G. (2019). Blood–brain barrier breakdown is an early biomarker of human cognitive dysfunction. Nat. Med..

[35] De Gregorio E., Del Franco M., Martinucci M., Roscetto E., Zarrilli R., Di Nocera P.P. (2015). Biofilm-associated proteins: News from Acinetobacter. BMC Genom..

[36] Noble J.M., Scarmeas N., Celenti R.S., Elkind M.S., Wright C.B., Schupf N., Papapanou P.N. (2014). Serum IgG antibody levels to periodontal microbiota are associated with incident Alzheimer disease. PLoS ONE.

[37] Pereira A.J.D.S.P.R., Tavares A.T., Prates M., Ribeiro N., Fonseca L.F., Marques M.D.R., Proença F. (2022). Brain abscess: A rare clinical case with oral etiology. Case Rep. Infect. Dis..

[38] Emery D.C., Shoemark D.K., Batstone T.E., Waterfall C.M., Coghill J.A., Cerajewska T.L., Davies M., West N.X., Allen S.J. (2017). 16S rRNA next generation sequencing analysis shows bacteria in Alzheimer’s post-mortem brain. Front. Aging Neurosci..

[39] Alonso R., Pisa D., Fernández-Fernández A.M., Carrasco L. (2018). Infection of fungi and bacteria in brain tissue from elderly persons and patients with Alzheimer’s disease. Front. Aging Neurosci..

[40] Ravaioli S., De Donno A., Bottau G., Campoccia D., Maso A., Dolzani P., Balaji P., Pegreffi F., Daglia M., Arciola C.R. (2024). The Opportunistic Pathogen *Staphylococcus warneri*: Virulence and Antibiotic Resistance, Clinical Features, Association with Orthopedic Implants and Other Medical Devices, and a Glance at Industrial Applications. Antibiotics.

[41] Leinonen R., Sugawara H., Shumway M. (2011). The Sequence Read Archive. Nucleic Acids Res..

[42] Afgan E., Baker D., Batut B., van den Beek M., Bouvier D., Cech M., Chilton J., Clements D., Coraor N., Grüning B.A. (2018). The Galaxy platform for accessible, reproducible and collaborative biomedical analyses: 2018 update. Nucleic Acids Res..

[43] Andrews S. (2010). FastQC: A Quality Control Tool for High Throughput Sequence Data.

[44] Bolger A.M., Lohse M., Usadel B. (2014). Trimmomatic: A flexible trimmer for Illumina sequence data. Bioinformatics.

[45] Wood D.E., Lu J., Langmead B. (2019). Improved metagenomic analysis with Kraken 2. Genome Biol..

[46] Lu J., Breitwieser F.P., Thielen P., Salzberg S.L. (2017). Bracken: Estimating species abundance in metagenomics data. PeerJ Comput. Sci..

[47] Ondov B.D., Bergman N.H., Phillippy A.M. (2011). Interactive metagenomic visualization in a Web browser. BMC Bioinform..

[48] Chong J., Liu P., Zhou G., Xia J. (2020). Using MicrobiomeAnalyst for comprehensive statistical, functional, and meta-analysis of microbiome data. Nat. Protoc..

[49] Robinson M.D., McCarthy D.J., Smyth G.K. (2010). edgeR: A Bioconductor package for differential expression analysis of digital gene expression data. Bioinformatics.

[50] Jumper J., Evans R., Pritzel A., Green T., Figurnov M., Ronneberger O., Tunyasuvunakool K., Bates R., Žídek A., Potapenko A. (2021). Highly accurate protein structure prediction with AlphaFold. Nature.

[51] Mirdita M., Schütze K., Moriwaki Y., Heo L., Ovchinnikov S., Steinegger M. (2022). ColabFold: Making protein folding accessible to all. Nat. Methods.

[52] Maurer-Stroh S., Debulpaep M., Kuemmerer N., Lopez de la Paz M., Martins I.C., Reumers J., Morris K.L., Copland A., Serpell L., Serrano L. (2010). Exploring the sequence determinants of amyloid structure using position-specific scoring matrices. Nat. Methods.

[53] Pettersen E.F., Goddard T.D., Huang C.C., Meng E.C., Couch G.S., Croll T.I., Morris J.H., Ferrin T.E. (2021). UCSF ChimeraX: Structure visualization for researchers, educators, and developers. Protein Sci..

