# Supplementary material for: Metatranscriptomic Reanalysis of Alzheimer’s Brains Identifies Low-Biomass Microbial Signals Including Enrichment of Acinetobacter radioresistens
Source: Int J Mol Sci. 2026 Apr 11;27(8):3430. doi: 10.3390/ijms27083430 (PMC13115908; doi:10.3390/ijms27083430)
Supplement: Supplementary file 1 [file ijms-27-03430-s001.zip › ijms-4202490-supplementary.pdf]

Supplementary Figure S1. Additional top-ranked taxa from the edgeR differential abundance analysis. Top-ranked microbial taxa identified in the species-level edgeR comparison between Alzheimer's disease (AD) and control samples that did not meet the **FDR < 0.10** trend threshold. Positive log2FC values indicate enrichment in AD, whereas negative values indicate enrichment in controls. These results are shown for completeness only.

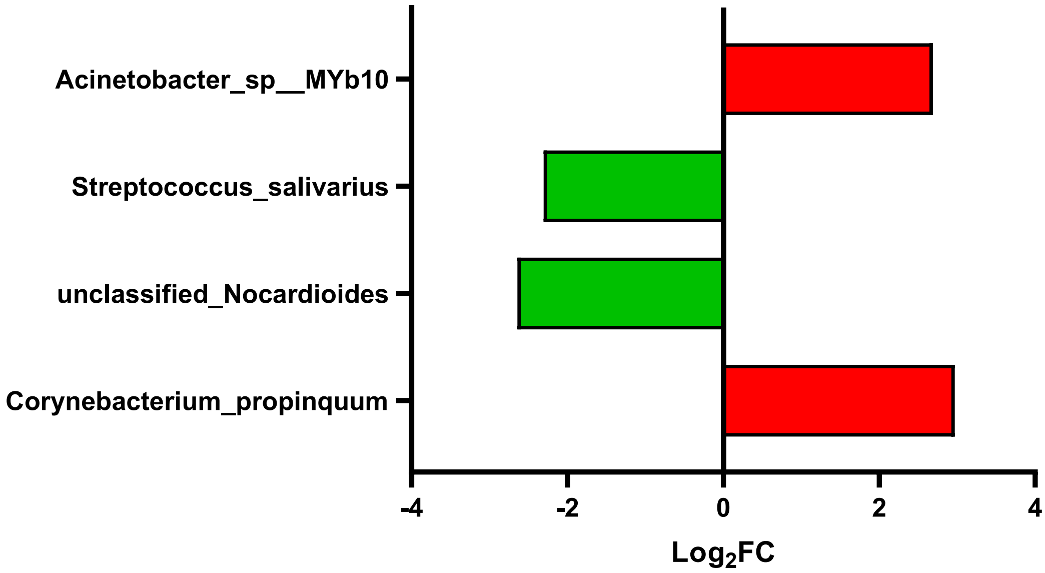

Supplementary Table S1: top-ranked taxa not meeting the FDR < 0.10 trend threshold

Bacteria	log2FC	P-value	FDR
***Acinetobacter* sp. MYb10**	2.6904	2.36×10^{-3}	0.10408
Streptococcus salivarius	-2.3041	2.68×10^{-3}	0.10408
**unclassified *Nocardioides***	-2.6423	3.09×10^{-3}	0.10697
Corynebacterium propinquum	2.9721	3.45×10^{-3}	0.10742

Supplementary Table S1. Additional top-ranked taxa from the species-level edgeR differential abundance analysis. The table lists the next-ranked microbial taxa identified in the Alzheimer's disease (AD) versus control comparison that did not meet the **FDR < 0.10** trend threshold. Positive log2FC values indicate relative enrichment in AD, whereas negative values indicate relative enrichment in controls.

Supplementary Table S2. Sample-level raw Bracken counts and group-wise prevalence for the top-ranked taxa identified in the differential abundance analysis. Raw Bracken counts are shown for each control (CTL_1–CTL_8) and Alzheimer's disease sample (AD_1–AD_9). Group-wise prevalence is reported as the number of samples with non-zero counts over the total number of samples in each group. This table is provided to facilitate assessment of sparsity, prevalence, and sample-level count distribution in this low-biomass dataset.

Taxon	CTL_1	CTL_2	CTL_3	CTL_4	CTL_5	CTL_6	CTL_7	CTL_8	AD_1	AD_2	AD_3	AD_4	AD_5	AD_6	AD_7	AD_8	AD_9	CTL prevalence	AD prevalence
**Acinetobacter radioresistens**	2	1	3	6	1	0	0	1	0	0	4	1754	2	22	2	0	3	6/8	6/9
**Lactobacillus iners**	0	57	3	2	0	175	136	0	0	0	0	10	5	4	1	0	2	5/8	5/9
**unclassified Arthrobacter**	4	1	12	0	22	306	0	5	1	0	0	7	1	0	1	0	1	6/8	5/9
**unclassified Actinomyces**	2	3	10	9	9	21	0	4	8	0	2	4	0	1	6	0	942	7/8	6/9
**unclassified Acinetobacter**	1	8	7757	24	2	109	0	14	8	34	10	21	15	24	7	0	25	7/8	8/9
**Staphylococcus warneri**	12	4	18	0	0	8	0	0	14	223	6	3	0	0	2	0	21	4/8	6/9
**Acinetobacter sp. MYb10**	1	2	6	11	0	0	0	0	1	34	3	8	14	5	1	0	4	4/8	8/9
**Streptococcus salivarius**	29	6	28	36	18	4	0	0	0	0	0	9	0	0	2	0	0	6/8	2/9
**unclassified Nocardioides**	0	2	242	3	52	17	0	4	0	0	0	0	0	3	0	0	16	6/8	2/9
**Corynebacterium propinquum**	1	1	6	9	0	6	0	2	0	52	3	0	0	1	0	2	39	6/8	5/9
